# Hypoxia-Induced Autophagy Enhances Cisplatin Resistance in Human Bladder Cancer Cells by Targeting Hypoxia-Inducible Factor-1*α*

**DOI:** 10.1155/2021/8887437

**Published:** 2021-02-17

**Authors:** Xiawa Mao, Nanzhang, Jiaquao Xiao, Huifeng Wu, Kefeng Ding

**Affiliations:** ^1^Urology Department, The 2nd Affiliated Hospital of Zhejiang University, School of Medicine, Hangzhou, Zhejiang Province 310000, China; ^2^Oncology Department, The 2nd Affiliated Hospital of Zhejiang University, School of Medicine, Hangzhou, Zhejiang Province 310000, China

## Abstract

**Purpose:**

To investigate the effect of hypoxia on chemoresistance and the underlying mechanism in bladder cancer cells.

**Methods:**

BIU-87 bladder cancer cell line was treated with cisplatin under hypoxic and normoxic conditions and tested using 3-(4,5-dimethylthiazol-2-yl)-2,5-diphenyltetrazolium bromide (MTT) assay, flow cytometry, and Western blotting. All the data were expressed as mean ± standard error from three independent experiments and analyzed by multiple *t*-tests.

**Results:**

Apoptosis of bladder cancer cells caused by cisplatin was attenuated in hypoxic conditions. Hypoxia enhanced autophagy caused by cisplatin. The autophagy inhibitor and HIF-1*α* inhibitor can reverse the chemoresistance in hypoxic condition. Apoptosis and autophagy of bladder cancer cells were downregulated by HIF-1*α* inhibitor YC-1. Hypoxia-induced autophagy enhanced chemoresistance to cisplatin via the HIF-1 signaling pathway.

**Conclusion:**

Resistance to cisplatin in BIU-87 bladder cancer cells under hypoxic conditions can be explained by activation of autophagy, which is regulated by HIF-1*α*-associated signaling pathways. The hypoxia–autophagy pathway may be a target for improving the efficacy of cisplatin chemotherapy in bladder cancer.

## 1. Introduction

Bladder cancer is the second most common genitourinary tumor and the fifth most common cause of cancer-related deaths in men in western countries [1]. Platinum-based systemic chemotherapy is important in the treatment of bladder cancer [2]. However, the general outcome is still unsatisfactory. Drug resistance continues to be a pivotal obstacle that limits the ideal therapeutic effects in patients with bladder cancer. In recent decades, much work has been done to attenuate the drug resistance and develop novel treatments that are less susceptible to resistance [3]. The mechanism for drug resistance can be ascribed to various factors, including altered drug sites, more effective DNA repair mechanisms, drug efflux and metabolism, pharmacokinetics, tumor microenvironment, and stress-induced genetic or epigenetic alterations in response to drug exposure [4]. Among the above reasons, hypoxic tumor microenvironment has to be taken into consideration. Carcinogenesis is closely related to the hypoxic environment within tumor tissues. Hypoxia-inducible factor- (HIF-) 1*α*, the most elevated protein stimulated by lack of oxygen, is widely expressed in tissues and cells [5]. According to recent literature, chemoresistance is largely due to expression and activation of HIF-1*α* [6].

Autophagy occurs extensively in eukaryotes and is an evolutionarily conserved process in which intracellular proteins and organelles are sequestered in autophagosomes, which are specialized double-membrane-containing vacuoles [7]. Through autophagy, tumors can counteract different kinds of adverse conditions, including hypoxia [8]. Under starvation or hypoxic conditions, cells are capable of eliminating excessive proteins and organelles, which may maintain homeostasis and facilitate cycling and reutilization of amino acids. It has been proved that HIF-1*α* has an intimate relationship with intracellular autophagy caused by a shortage of oxygen. HIF-1*α* can regulate hypoxia-induced autophagy by manipulating expression of downstream targeting genes BNIP3 and BNIP3L, inducing autophagy by interfering with the interaction between Beclin-1, Bcl-2, and BCL-XL [9, 10].

Bladder urothelial carcinoma is an active metabolic tumor with a higher oxygen consumption than healthy tissue has. As a result, the tumor tissue is partially in a hypoxic environment. However, little work has been performed on the relationship between chemoresistance and hypoxia in bladder cancer. Here, we investigated the influence of low oxygen microenvironment on chemoresistance to cisplatin in bladder cancer cells and tried to explain the underlying mechanism. This study is aimed at revealing how hypoxia and autophagy interact with each other to mediate cisplatin resistance in bladder cancer cells.

## 2. Materials and Methods

### 2.1. Cell Culture

The BIU-87 bladder cancer cell line was purchased from Kunming Cell Bank, Chinese Academy of Sciences. BIU-87 cells were initially cultured in RPMI 1640 medium (Gibco, CA, USA) and incubated with 10% fetal bovine serum (Cyclones, Logan, UT, USA) and penicillin/streptomycin (Gibco) at 37°C in 5% CO_2_ atmosphere.

### 2.2. Ethics Statement

Human tissues and samples were not required in this study. All work presented has been performed in well-established, commercially available cell lines.

### 2.3. Treatment of Cells

BIU-87 cells were treated with different concentrations of cisplatin (Sigma–Aldrich, St. Louis, MO, USA) for hours or days to perform the 3-(4,5-dimethylthiazol-2-yl)-2,5-diphenyltetrazolium bromide (MTT) assay. These cells were grown in 20% or 1% O_2_ and 5% CO_2_ atmosphere, respectively. To observe the effect of 3-methyladenine (3-MA; Selleck Chemicals, Houston, TX, USA) or lificiguat (YC-1; Selleck Chemicals), BIU-87 cells were first cultured in 96-well or 6-well plates for 24 h and then cisplatin and inhibitors were added for a further 24 h. These cells were cultured in 20% or 1% O_2_ and 5% CO_2_ atmosphere in hypoxic incubator (Thermo Fisher 3111). Samples were collected and analyzed by Western blotting, as described below.

### 2.4. MTT Assay

Cell viability after treatment was investigated by MTT assay. Cells (5000/well, 100 *μ*l medium) were cultured in a 96-well plate (Corning, Corning, NY, USA), and after treatment, 20 *μ*l MTT was added (5 mg/mL in PBS) and incubated for 4 h at 37°C. We removed the medium and MTT solution, added 100 *μ*l dimethyl sulfoxide (Sigma–Aldrich), and measured at 490 nm by ELISA Plate Readers (BioTek, Winooski, VT, USA).

### 2.5. Flow Cytometry

Cells after treatment were collected and washed in PBS 3 times. They were resuspended in loading buffer (Sangon Biotech, Shanghai, China) to 2 × 105 to 5 × 105 cells/mL. For apoptosis and autophagy analysis, Annexin V-FITC/PI kit (Sangon Biotech) and monodansylcadaverine (MDC) kit (Beijing Solarbio Science & Technology Co. Ltd., Beijing, China) were utilized, respectively. BD FACSCanto™ II system (BD Biosciences, San Jose, CA, USA) was utilized to detect cell surface markers. All the results were analyzed by the FlowJo software.

### 2.6. MDC Fluorescence Staining

BIU-87 cells grown on coverslips were used for MDC fluorescence staining. The cells were cultured in 20% or 1% O_2_ and 5% CO_2_ atmosphere. Coverslips were soaked in 75% ethanol overnight and washed in PBS (Sangon Biotech) 3 times. The coverslips were put in 6-well plates (Corning), and cell suspension was added. One day later, we added cisplatin and inhibitors to the cells for a further day and removed the coverslips with the cells and washed in PBS 3 times. Cells were fixed with 4% paraformaldehyde (Sangon Biotech) for 30 min at room temperature and washed in PBS 3 times. The cells were incubated with 10% MDC for 30 min and washed in PBS 3 times. All the images were photographed by Leica DM4000B microscope (Leica, IL, USA).

### 2.7. Western Blotting

Cells were lysed in RIPA buffer (Beyotime Biotechnology, Shanghai, China) containing protease and phosphatase inhibitor cocktails (Thermo Fisher Scientific, Waltham, MA, USA). The protein concentration of the samples was quantified by the BCA assay kit (Beyotime Biotechnology), and equal amounts of protein were loaded on 10% SDS-PAGE and transferred to polyvinylidene difluoride membrane (Millipore, Billerica, MA, USA). The membranes were blocked with 5% nonfat milk for 1 h and incubated with primary antibody overnight at 4°C. The following primary antibodies were used to detect proteins: rabbit anti-Beclin-1 polyclonal antibody (1 : 500; Affinity, Canal Fulton, OH, USA), rabbit anti-ATG 5 polyclonal antibody (1 : 500, Affinity, OH, USA), mouse anti-HIF-1A monoclonal antibody (1 : 500; Affinity), and rabbit anti-caspase-3 polyclonal antibody (1 : 500; Proteintech, Wuhan, China). The membranes were incubated with a goat anti-rabbit IgG conjugated to horseradish peroxidase (1 : 1000; Beyotime) or a goat anti-mouse IgG conjugated to horseradish peroxidase (1 : 1000; Beyotime) for 1 h. Incubation with a mouse anti-*β*-actin monoclonal antibody (1 : 1000; Abcam, Cambridge, UK) was performed as the loading sample control. The blots were detected using Clarity MAX™ Western ECL Substrate (BioRad, Hercules, CA, USA) by ChemiDoc™ XRS+ System (BioRad).

### 2.8. Statistical Analysis

All the data were expressed as mean ± standard error from three independent experiments and analyzed by multiple *t*-tests. Data were graphically displayed using GraphPad Prism version 6.0 (GraphPad Software, La Jolla, CA, USA). A *p* value <0.05 was considered statistically significant for all the analyses.

## 3. Results

### 3.1. Hypoxia Induced Chemoresistance in BIU-87 Bladder Cancer Cells

**Figure 1:**
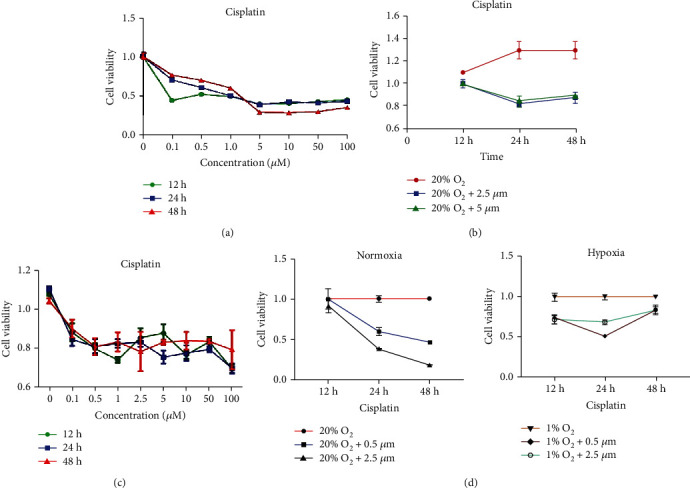
Hypoxia reduced chemosensitivity of bladder cancer cells to cisplatin. (a) Viability of bladder cancer cells was measured after treatment with graded concentrations of cisplatin at 12, 24, and 48 h. (b) Cell viability was further observed after treatment with 2.5 and 5.0 *μ*m cisplatin under normoxia. (c) BIU-87 cancer cells were cultured under hypoxic conditions with different concentrations of cisplatin for 12, 24, and 48 h. (d) Viability of cells incubated with cisplatin was reversed under hypoxia compared with that under normoxia.

Resistance to chemotherapeutic agents is a pivotal obstacle that is encountered in the chemotherapy of urological tumors [11]. To explore the exact mechanisms behind this resistance, we designed experiments to observe the difference in cell growth in normoxic and hypoxic conditions, which were defined as 5% CO_2_ with 20% O_2_ and 5% CO_2_ with 1% O_2_, respectively. BIU-87 cancer cells were maintained in complete medium under normoxia and hypoxia and incubated with graded concentrations of cisplatin. At 12 h, the cell proliferation rate was decreased by cisplatin, but the trend was not apparent with the increase in drug concentration. At 24 and 48 h, the cell inhibition rate was increased in a dose-dependent manner after cisplatin treatment. The trend became more evident at concentrations above 5 *μ*M cisplatin and influencing cell proliferation (Figure 1(a)). Due to the significant change in inhibition rates, the concentration refinement experiments at 2.5 *μ*M and 5 *μ*M were further performed. The inhibition rates of BIU-87 cells were similar between 2.5 and 5 *μ*M cisplatin (Figure 1(b)). Therefore, 2.5 *μ*M was selected for the next experiment.

The inhibition rate of cisplatin fluctuated about 20% with increase in concentration when BIU-87 cells were cultured under hypoxic conditions (Figure 1(c)). However, little changes at 100 *μ*M concentration and irregular changes at 12 h were observed simultaneously. With the same concentration of cisplatin, the inhibition rate of BIU-87 cells under hypoxia was significantly downregulated compared with that under normoxia (Figure 1(d)), indicating that cisplatin resistance may have been induced by hypoxia in BIU-87 cells.

### 3.2. Flow Cytometry Verified Chemoresistance of BIU-87 Cells to Cisplatin

**Figure 2:**
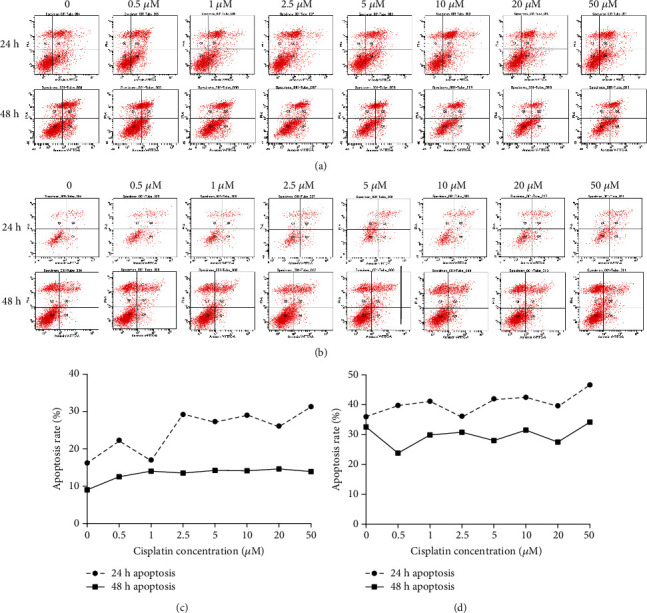
Effects of cisplatin on apoptosis of bladder cancer cells under normoxic and hypoxic conditions. (a, c) Under normoxia, the apoptosis rate was significantly increased with increase of drug concentration, from 16.2% to 31.3% at 24 h and 9% to 14.6% at 48 h. (b, d) Under hypoxia, the apoptosis rate was slightly increased with increase of drug concentration, from 36% to 46.7% at 24 h and 32.6% to 34.2% at 48 h.

Cisplatin-induced death in bladder cancer cells involves multiple signaling pathways. Among these pathways, mitochondrial apoptosis plays a central role. The MTT assay inferred that chemotherapeutic agents and hypoxia have definite effects on the viability of BIU-87 cells. To elucidate the cause of this phenomenon, we investigated the effects of drugs on apoptosis. Flow cytometry demonstrated that apoptosis rate was significantly increased (16.2–31.3% at 24 h to 9–14.6% at 48 h) with increase of drug concentration under normoxia. In contrast, the apoptosis rate increased slightly with increase of drug concentration (36–46.7% at 24 h to 32.6–34.2% at 48 h) under hypoxia. After 48 h treatment, the apoptosis rate was not significantly different from that under normoxia. After treatment with 2.5 *μ*M cisplatin at 24 h, the apoptosis rate was higher than that of control cells under normoxic conditions (28.6% vs. 16.2%) (Figures 2(a) and 2(c)). However, a similar change was not detected under hypoxic conditions (36% vs. 36.7%). This suggests that drug toxicity to cancer cells under normoxia is enhanced with increase of drug concentration, and such an effect is impaired by hypoxia (Figures 2(b) and 2(d)). The results at 48 h may be attributed to drug deactivation at this point.

### 3.3. Effects of Autophagy Inhibitor and HIF-1 Inhibitor on Chemoresistance of BIU-87 Cells under Normoxia and Hypoxia

**Figure 3:**
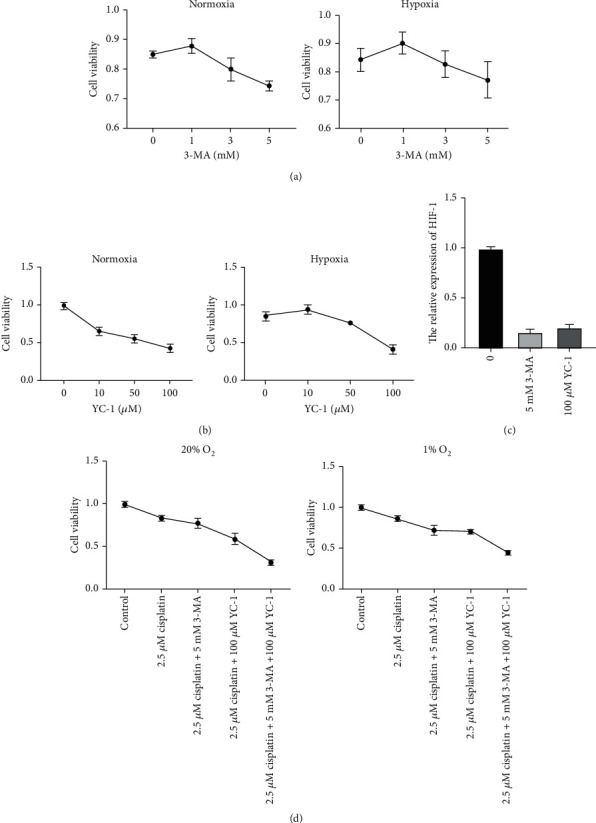
Effect of autophagy inhibitor and HIF-1 inhibitor on BIU-87 bladder cancer cell chemoresistance under hypoxia and normoxia. (a) Under normoxic and hypoxic, toxicity of cisplatin in BIU-87 cells was increased with the increased concentration of 3-MA. (b) Under normoxic and hypoxic, toxicity of cisplatin in BIU-87 cells was increased with increased concentration of YC-1. (c) The relative expression of HIF-1 in BIU-87 cells treated with 100 *μ*M YC-1. (d) Under normoxic and hypoxic conditions, viability of BIU-87 cells was observed in different groups.

The above results proved that hypoxia induced resistance to chemotherapy, and apoptosis was related to this procedure. It is reported that cancer cells make themselves more adaptive through autophagy in adverse conditions. Therefore, we hypothesized that autophagy played an essential role in this process. To test this hypothesis, we inhibited autophagy by 3-MA and HIF-1 by YC-1 under hypoxic and normoxic conditions. According to the MTT assay, after incubation with 3-MA and HIF-1*α* inhibitor YC-1, cisplatin cytotoxicity was strengthened with increased concentration of 3-MA and YC-1 (Figures 3(a) and 3(b)). We chose 5 mM 3-MA and 100 *μ*m YC-1 for the following experiments.

In order to investigate the effect of 3-MA and YC-1, we examined the expression of HIF-1*α* by qRT-PCR. The results showed that under hypoxia, 5 mM 3-MA and 100 *μ*m YC-1 could inhibit the expression of HIF-1*α* effectively in BIU-87 cells (Figure 3(c)). Under normoxic conditions, inhibition of the autophagy pathway could decrease cell activity. This environment causes autophagy and thus shows resistance to cisplatin. To verify the above assumption, we continued the relevant tests under hypoxic conditions. The trend under hypoxia was similar to that under normoxia (Figure 3(d)). It is worth noting that this trend under hypoxia was less than that under normoxia (relative viability of cells under normoxic conditions, cisplatin: 83.9%, cisplatin+3-MA: 77.0%, cisplatin+YC-1: 59.2%, and cisplatin+3-MA+YC-1: 31.5%; relative viability under hypoxic conditions, cisplatin: 86.0%, cisplatin+3-MA: 72.5%, cisplatin+YC-1: 71.0%, and cisplatin+3-MA+YC-1: 45.2%). This result suggests that autophagy and the HIF-1*α* gene are likely involved in the hypoxic resistance to cisplatin in BIU-87 cancer cells.

### 3.4. Autophagy Analysis in the Drug Resistance Process

**Figure 4:**
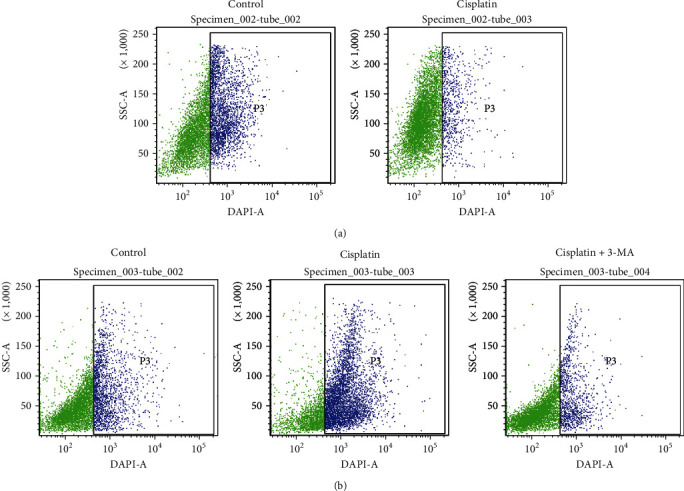
Autophagy analysis of BIU-87 bladder cancer cells. (a) Under normoxia, flow cytometry revealed that autophagy was not activated in the cisplatin group (8%) compared with the control group (27.7%). (b) Under hypoxia, flow cytometry showed that autophagy was 19.4% in the control group, significantly increased in the cisplatin group (48.1%) and downregulated after incubation with autophagy inhibitor 3-MA.

MDC is a fluorescent compound that is incorporated into multilamellar bodies by an ion-trapping mechanism and interaction with membrane lipids and is a probe for detection of autophagic vacuoles in cultured cells [12]. Flow cytometry showed that the control group had 27.7% autophagy while the cisplatin group had only 8% under normoxic conditions, suggesting that the cells could not undergo autophagy under normoxic conditions to counteract drug toxicity (Figure 4(a)). Compared with the control group (19.4%), autophagy was increased significantly (48.1%) in the drug group and dramatically reduced after addition of 3-MA (11.3%). This indicated that drug resistance of the cells in the hypoxic environment was indeed achieved through autophagy regulation (Figure 4(b)).

The above results proved that under hypoxic conditions, cisplatin induced autophagy in cancer cells, which was in accordance with the results of MDC detection.

### 3.5. Autophagy and Apoptosis Were Involved in Chemoresistance to Cisplatin under Hypoxic Conditions as Proved by Molecular Tests

The MTT assay (Figure 2) showed that apoptosis was involved in drug resistance. To confirm this, we designed experiments to detect several major marker proteins and genes related to the process.

**Figure 5:**
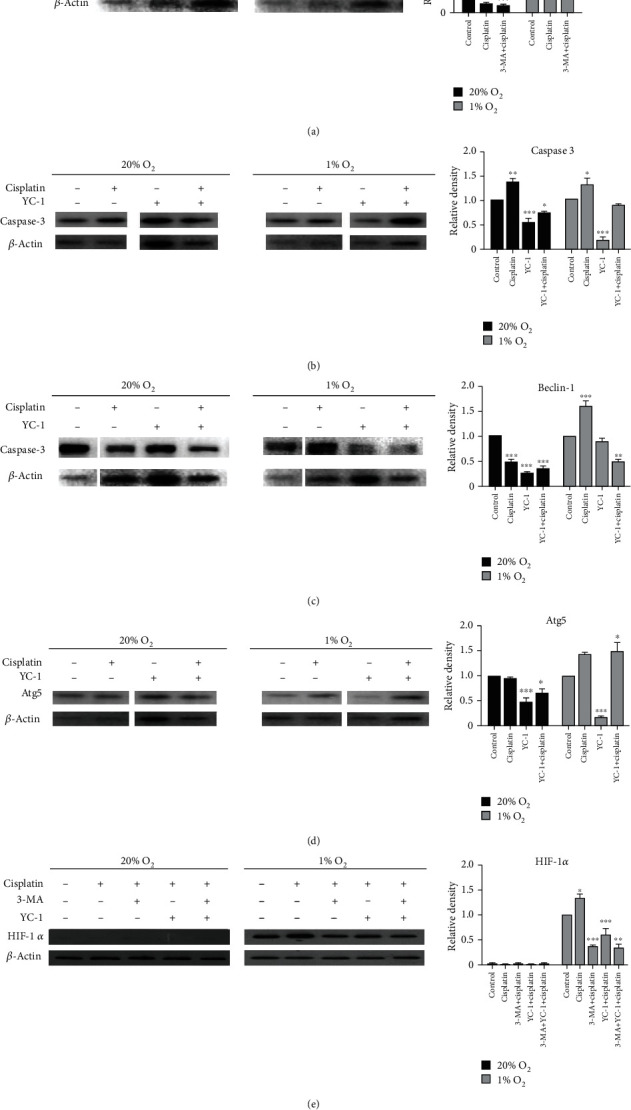
Expression of related marker proteins and genes under normoxic and hypoxic conditions by Western blotting. (a) Beclin-1 was measured by immunoblotting under normoxia and hypoxia. (b) Caspase-3 was measured by immunoblotting when incubated with HIF-1*α* inhibitor YC-1. The results indicate that hypoxia might induce cell chemoresistance through the HIF-1*α* signaling pathway. (c) Beclin-1 was measured by immunoblotting under normoxia and hypoxia. (d) Atg5 was measured by Western blotting and showed a similar trend to caspase-3. (e) HIF-1*α* was measured with Western blotting. The result revealed that HIF was involved in targeting apoptosis induced by autophagy.

Beclin-1 is used as a marker protein of cell autophagy. Under normoxia, expression of Beclin-1 in the cisplatin group was lower than that in the control group and similar to that in the autophagy inhibitor group (Figure 5(a)). In contrast, expression of Beclin-1 under hypoxia was significantly increased after treatment with cisplatin and obviously decreased after incubation with 3-MA. These results were similar to those of the MTT assay and MDC test, suggesting that hypoxia promotes autophagy, making cells more adaptive to chemotherapy.

Under normoxia, expression of caspase-3 was downregulated after treatment with YC-1 and the downward trend was alleviated when followed by cisplatin. Under hypoxic conditions, expression of caspase-3 did not change much in the study group compared with that in the control group, indicating that apoptosis in the two groups was similar (Figure 5(b)). This result demonstrated that hypoxia might induce drug resistance in cells by targeting the HIF-1*α* signaling pathway.

Under hypoxia, expression of Beclin-1 was significantly upregulated after treatment with cisplatin, downregulated by YC-1, and lowest after treatment with both agents (Figure 5(c)). This suggests that hypoxia may cause autophagy in cells, and decreased expression of HIF-1*α* may attenuate autophagy. This suggests that a lower oxygen environment produces cell autoregulation by targeting HIF-1*α* expression. This pathway is responsible for drug resistance.

Atg5, a molecule involved in autophagy, is known to be regulated via various stress-induced transcription factors and protein kinases [13]. Expression of Atg5 was similar to that of caspase-3 (Figure 5(d)), supporting the hypothesis at the molecular level that hypoxic conditions may cause autophagy in cells by targeting HIF-1*α*.

Based on previous results, HIF-1*α* has been confirmed to be involved in this process. Consequently, it is necessary to investigate how it changes and works. Expression of HIF-1*α* could not be detected under normoxia. It was upregulated under hypoxia, significantly downregulated after adding 3-MA and cisplatin, and similarly reduced after adding YC-1 and cisplatin compared with that in the negative control group (Figure 5(e)). We conclude that cisplatin may induce apoptosis in BIU-87 cells under hypoxic conditions by targeting HIF-1*α*-mediated autophagy.

## 4. Discussion

Hypoxia has been proved as one of the important factors in promoting chemoresistance of cancers cells. Autophagy has been extensively studied as a cytoprotective mechanism against chemotherapy [14]. During the past decade, there has been an increasing interest in the relationship between hypoxia and autophagy [15, 16]. However, little work has been performed on bladder cancer [17]. The present study demonstrated that cisplatin was lethal to BIU-87 cells under normoxic conditions, but hypoxia reversed the cytotoxicity of chemotherapy by mediating autophagy through targeting HIF-1*α*.

It has been reported that cancer cells undergo a series of complex molecular changes that allow them to be more adaptive to chemotherapeutic agents [18–20]. Similar to this, our study showed that cisplatin was less toxic to bladder cancer cells under hypoxic compared with normoxic conditions. One of the principal factors attributed to the difference is HIF [21, 22]. Researches have proved that HIF-1*α* is involved in hypoxia-induced chemoresistance [23–25]. Our results supported the finding that hypoxia decreased sensitization to cisplatin in bladder cancer cells by targeting apoptosis. We also showed that HIF-1*α* was hardly expressed under normoxia but highly upregulated under hypoxia and downregulated when treated with HIF inhibitor YC-1. The underlying mechanisms of hypoxia-induced chemoresistance are manifold, including MDR gene expression, reduced reactive oxygen species levels, cell cycle arrest, gene mutations, and drug concentration decreases [26–30].

Autophagy is one of the decisive factors that assist cancer cells to counteract various adverse conditions. Hypoxia can activate autophagy and hypoxia-induced autophagy may protect cells from apoptosis induced by chemotherapeutic agents [31–34]. Consistent with the above reports, our study demonstrated that MDC and Beclin-1 were increased under hypoxia, and simultaneously, bladder cancer cells were less sensitive to cisplatin. However, when incubated with 3-MA, a popular inhibitor of autophagy that inhibits conversion of LC3-I to LC3-II by targeting PI3K [34], hypoxia-induced autophagy was significantly attenuated, suggesting that autophagy induced under hypoxic conditions contributes to chemoresistance of bladder cancer. Therefore, 3-MA can be used to enhance apoptosis when combined with chemotherapeutic agents under hypoxic conditions [35, 36]. When incubated with YC-1, a typical HIF inhibitor, caspase-3 was upregulated under hypoxic conditions, indicating that the regulatory effect of cisplatin on apoptosis may be achieved by targeting the HIF-1*α* signaling pathway. Furthermore, expression of Beclin-1 and Atg5 showed no change under normoxia, increased under hypoxia, and was downregulated when treated with YC-1 sequentially, suggesting that autophagy induced by hypoxia may be achieved by regulating expression of HIF-1*α*. Sun et al. thought HIF-1*α*/MDR1 pathway confers the chemoresistance to cisplatin in bladder cancer [37]. Combined with our results, it indicates that a variety of mechanisms are involved in the chemoresistance of bladder cancer. Future study could be focused on combined experiments of HIF-1a/MDR1 and autophage.

It should be noted that our study examined only one bladder cancer cell line and a single chemotherapeutic agent. Future research should focus on multiple bladder cancer cell lines and more chemotherapeutic drugs. Additionally, in vivo experiments will make the results more convincing and acceptable.

## 5. Conclusions

To sum up, the present study revealed that resistance to cisplatin in BIU-87 bladder cancer cells under hypoxic conditions could be explained by the activation of autophagy, which was regulated by HIF-1*α*-associated signaling pathways. Accordingly, the hypoxia–autophagy pathway may be a target for improving the efficacy of cisplatin chemotherapy in bladder cancer.

## Data Availability

The data used to support the findings of this study are included within the article.
